# Spatial-Temporal Analysis and Driving Factors Decomposition of (De)Coupling Condition of SO_2_ Emissions in China

**DOI:** 10.3390/ijerph17186725

**Published:** 2020-09-15

**Authors:** Yue Wang, Lei Shi, Di Chen, Xue Tan

**Affiliations:** 1 School of Environment and Natural Resources, Renmin University of China, Beijing 100872, China; wangyue_prc@ruc.edu.cn (Y.W.); chendi16@ruc.edu.cn (D.C.); 2 Division of the Social Science, The University of Chicago, Chicago, IL 60615, USA; 3 State Grid Energy Research Institute Co., LTD, Beijing 102209, China; tanxue@sgeri.sgcc.com.cn

**Keywords:** decoupling analysis, driving factors decomposition, Moran Index, generalized logarithmic mean Divisia index, SO_2_ emissions, China

## Abstract

China has a fast-growing economy and is one of the top three sulfur dioxide (SO_2_) emitters in the world. This paper is committed to finding efficient ways for China to reduce SO_2_ emissions with little impact on its socio-economic development. Data of 30 provinces in China from 2000 to 2017 were collected to assess the decoupling relationship between economic growth and SO_2_ emissions. The Tapio method was used. Then, the temporal trend of decoupling was analyzed and the Moran Index was introduced to test spatial autocorrelation of the provinces. To concentrate resources and improve the reduction efficiency, a generalized logarithmic mean Divisia index improved by the Cobb–Douglas function was applied to decompose drivers of SO_2_ emissions and to identify the main drivers. Results showed that the overall relationship between SO_2_ emissions and economic growth had strong decoupling (SD) since 2012; provinces, except for Liaoning and Guizhou, have reached SD since 2015. The decoupling indexes of neighboring provinces had spatial dependence at more than 95% certainty. The main positive driver was the proportion of the secondary sector of the economy and the main negative drivers were related to energy consumption and investment in waste gas treatment. Then, corresponding suggestions for government and enterprises were made.

## 1. Introduction

Since the 21st century, China’s rapid economic growth and social development has given rise to a series of environmental problems. Air pollution has been identified as a high priority issue that must be addressed immediately. Among air pollutants, SO_2_ has aroused wide concern from all parts of society. From an international perspective, SO_2_ emission issues in China are still serious. In 2018, China’s SO_2_ emissions were 2578 thousand tons; it was the third highest in the world, just behind India and Russia (http://sputniknews.cn/economics/201908191029324285/). SO_2_ emissions will not only lower the atmospheric quality, but also trigger health-related issues [1]. A 10μg/m^3^ increase in SO_2_ emissions was associated with an increase in out-of-hospital coronary deaths (of 0.88%) [2]. A significant long-term relationship between SO_2_ and end-stage renal disease (ESRD)-related mortality was also found [3]. Therefore, it is essential for China to unceasingly reduce emissions of SO_2_ while developing the economy. To achieve this “win-win” condition between economic growth and SO_2_ emissions, the relationship between economic development and SO_2_ emissions should be studied intensively and deeply.

Decoupling is defined as the break of a coupling relationship between economic development and environmental quality [4]. When environmental pollution no longer increases with the growth of the economy, the decoupling condition occurs. The idea of decoupling “environmental bads” from “economic goods” has been proposed as a path towards sustainability by organizations, such as the Organization for Economic Co-operation and Development (OECD) [5] and the United Nations (UN) [6]. Decoupling analysis is an academic theory to study whether economic and environment-related indicators have decoupled [6]. At the end of the 20th century, academic researches using decoupling analysis started to develop after Weizsäcker’s study on economic growth and resource consumption [7]. Nowadays, the decoupling analysis has been widely used to test the dependence of economic growth on air pollutant emissions. Wu et al. [8] accomplished the decoupling analysis in China at provincial level, and then they used social network analysis (SNA), quadratic assignment procedure (QAP) regression, and logit model to further study the spatial characters of decoupling. The results showed that the economically developed eastern coastal areas had better decoupling conditions than less economically developed regions, such as Xinjiang, Hainan, Qinghai, and Tibet. The temporal change of each province had not been presented due to the limitation of spatial approaches. Zhang et al. [9] completed China’s decoupling analysis between PM_2.5_ emissions and economic growth, and then they further analyzed the trend of decoupling index and its driving factors. The results showed that China’s PM_2.5_ emissions were weakly decoupled with economic growth. Possible spatial associations between different provinces were not covered due to the limitation of temporal approaches. Obviously, after decoupling analysis, scholars had researched further through various methods. However, few studies have combined temporal and spatial analysis to fully study decoupling conditions between economic growth and air pollutant emissions in China. With China’s socio-economic development, the decoupling conditions in provinces are likely to change significantly. To deeply study the dynamic trend and stability of decoupling conditions, analysis from the temporal perspective is necessary. At the same time, considering that the economic development of each province may be affected by neighboring provinces, and the SO_2_ emission of each province may also affect neighbors by the atmospheric flow; the decoupling index of each province may be spatially related. There can be essential reference to policy-making and coordinated development of provinces if such spatial autocorrelation existed. Therefore, it is necessary to add spatial analysis to the research. To provide more comprehensive references for decision makers, spatial-temporal analysis was adopted in this research, which uses trend analysis and spatial correlation analysis to study decoupling conditions from both temporal and spatial aspects [10].

Researchers have found that the reduction of SO_2_ emissions in China can be costly. The mean values of SO_2_ abatement cost for every year ranged from 13,765 RMB yuan per ton to 14,711 RMB yuan per ton [11]. Thus, it is vital to further improve the efficiency of SO_2_ emissions reduction in China. Nevertheless, the understanding of decoupling conditions alone cannot maximize the efficiency of SO_2_ reduction, because SO_2_ emissions are affected by various drivers, such as demographic factors [12], energy factors [13], economic factors [14], and so on. Driving factors decomposition is a method used to decompose the effect of each driving factor from the total effect on SO_2_ emissions [15]. By doing this, different factors’ impact on SO_2_ emissions can be analyzed; thus, the main driving factors of SO_2_ emissions can be identified. Concentrating resources to deal with these main drivers can not only improve the effect of emission reduction, but also cut down the expenditures. The logarithmic mean Divisia index (LMDI) proposed by Ang et al. [16] is one of the mainstream methods in exponential decomposition analysis, due to advantages, including zero residual, path independency, and consistency in aggregation [17]. Moreover, LMDI is often used as a supplement of the decoupling analysis [18]. However, the influence of factors, such as capital and labor, are not considered in the LMDI method, which are important determinants of economic growth and may have impacts on SO_2_ emissions. Thus, in this paper, a generalized LMDI (GLMDI) method was constructed to make up for the omissions of the LMDI method; thus, the driving factors of SO_2_ emissions can be studied more comprehensively.

The purpose of this paper is to find efficient ways for China to reduce SO_2_ emissions with as little impact on its socio-economic development as possible. Therefore, this paper collected data of 30 provinces of China (excluding Hong Kong, Macao, Taiwan, and Tibet) from 2000 to 2017. Firstly, the annual decoupling index of each province was calculated for temporal analysis. The focus of this part was to analyze the dynamic change trend of decoupling conditions from both national and provincial perspectives. Secondly, spatial analysis was done by making comparisons among provinces over the entire time period, and by studying the spatial autocorrelation between neighboring provinces. The focus of this part was to compare the state of provinces over the whole time period and to find out the interaction patterns between neighboring areas. Thirdly, to decompose the effect of different driving factors comprehensively, this paper used the Cobb–Douglas (C–D) production function to improve the LMDI model. The C–D production function was introduced by Cobb and Douglas in 1928 [19], it takes labor and capital as the main factors to establish an exponential relationship model. Thus, using the C–D production function can make up for the lack of capital and labor in LMDI. Based on the C–D production function and the LMDI method, a GLMDI method was constructed, referring to research by Wang et al. [20]. Then, effects of 10 representative driving factors were decomposed by the GLMDI method; their impacts on SO_2_ emissions were discussed and compared. The task, of this part, was to analyze the influence of each driving factor on SO_2_ emissions; thus, the main drivers could be found out, which should be focused on by the Chinese government to improve the efficiency of SO_2_ emissions reduction.

The rest of this paper is organized as follows. Section “Materials and Methods” presents the research methods and relevant data sources. Section “Results and Discussion” analyzes the decoupling conditions from a spatial-temporal perspective and decomposes driving factors of SO_2_ emissions in China over years. The main conclusions of this paper are in Section “Conclusions”.

## 2. Materials and Methods

### 2.1. Tapio Elastic Analysis Method

As research on decoupling grows, various methods for determining and analyzing decoupling status has been developed, including the OECD decoupling factor method, the variation analysis (VA) method, and the Tapio elastic analysis (TEA) method. Although all three methods are workable, they have some limitations. The OECD decoupling factor model cannot distinguish the decoupling states in an expanding and recessive economy [21]; the VA method cannot distinguish between non-decoupling and re-decoupling [22]; and the TEA method defines much decoupling states. Wu et al. summarized these decoupling methods and compared their advantages and disadvantages; the result showed that the TEA model was more accurate and not limited by the length of time [23]. Therefore, this paper selects the TEA method to assess the decoupling relationship between economic growth and SO_2_ emissions in China. Based on the general concept of the TEA model, the decoupling elasticity index between SO_2_ Emissions and economic growth can be written as:

(1) $$e_{t+1}=\frac{\Delta S_{t+1}}{\Delta G_{t+1}}=\frac{\frac{S_{t+1}}{S_{t}}-1}{\frac{G_{t+1}}{G_{t}}-1}$$

where, $e_{t+1}$ denotes the decoupling index between year *t* and year *t +* 1. $\Delta S_{t+1}$, $\Delta G_{t+1}$ represent the change rate of SO_2_ emissions and economic growth from year *t* to year *t*+1 separately. $S_{t+1}$, $G_{t+1}$, respectively, denote SO_2_ emissions and economic growth in year *t +* 1 while $S_{t}$, $G_{t}$ denotes those conditions in year *t*. In this paper, the Gross Regional Product (GRP) of each province, converted to comparable prices based on the year 2000, was used to measure economic growth level. The data of provincial GRP figures, GRP indices, and SO_2_ emissions were derived from the National Bureau of Statistics of China.

On the basis of definition, classification, and empirical analysis mentioned by Tapio [24], the decoupling relationship between air pollutant emissions and economic growth can be divided into three states, namely, decoupling, coupling, and negative decoupling, which can be further subdivided into eight logical possibilities, as shown in Table 1. According to Tapio’s research [24], in order not to over-interpret slight changes as significant, ±20% variation of the $e_{t+1}$ values around 1.0 are still regarded, here, as coupling. Thus, coupling is defined as $e_{t+1}$ values from 0.8 to 1.2, which is an empiric value. On the other hand, the growth of the variables can be positive or negative, expressed as expansive coupling and recessive coupling.

### 2.2. Moran Index

At present, there are two commonly used methods to test spatial dependence, which are Geary’s coefficient [25] and the Moran Index (Moran’s I) test [26]. Both of them have limitations. The Geary’s coefficient could be sensitive to high values, which means that it has a better ability to detect high-value spatial clustering than low-value spatial clustering. The Moran’s I could be mainly affected by the size of the aggregation area, which means that it will increase with the expansion of the spatial clustering range. However, researchers have found that it is more reliable to use Moran’s I to judge whether there is spatial clustering in a region [27]. Our main purpose was to determine whether there is spatial correlation between provinces; the Moran’s I was adopted. The expression of Moran’s I is as follows:

(2) $$Moran\prime s\;I=\left[n\sum_{i=1}^{n}\sum_{j=1}^{n}\omega_{ij}\left(x_{i}-\bar{x}\right)\left(x_{j}-\bar{x}\right)\right]/\left[\sum_{i=1}^{n}\sum_{j=1}^{n}\omega_{ij}\sum_{i=1}^{n}{\left(x_{i}-\bar{x}\right)}^{2}\right]$$

where, *n* represents provinces in China. $x_{i}$*,* $x_{j}$ are the decoupling indicators from 2000 to 2017 of *i* province, *j* province, respectively. $\bar{x}$ denotes the mean of decoupling indexes. $\omega_{ij}$ denotes the spatial weight, which represents the strength of potential interaction between individual provinces. There are various approaches to generate a spatial weight matrix including queen contiguity [28], rook contiguity [29], distance weight [30], and k-nearest neighbors [31]. The matrixes generated by the first two methods are composed of 0 and 1. Rook contiguity stipulates that if two provinces have a common boundary, they are considered adjacent, and the corresponding value in the spatial weight matrix is 1, otherwise, the value in matrix is 0. Queen contiguity stipulates that if two provinces have a common boundary or a common point, they are considered adjacent. Within the scope of this study, Hainan has neither a common border nor a common point with other provinces. In order not to ignore the relationship between Hainan and other provinces, the first two methods were not adopted in this paper. The latter two methods construct the matrix according to the geographical distance between provinces. The k-nearest neighbors method is to calculate the distance between each province and its nearest *k* provinces, *k* is the number of neighbors, which needs to be set by scholars. In this method, each province has the same number of neighbors, which is subjective to some extent. For the distance weight theory, the threshold of a minimum space range is calculated to ensure that each province has at least one neighbor. Then, the distances between each province and its neighbors are calculated to generate the spatial weight matrix. This approach is more objective and can make the influence scope of each province consistent, so, this paper adopts distance weight matrix method. The provincial longitude and latitude data were derived from the National Bureau of Statistics of China.

Moran’s I ranges from −1 to 1. If it is greater than 0, there is positive spatial autocorrelation. The larger the value, the stronger the positive spatial dependence. If the index is less than 0, there is no similar attributes between adjacent provinces, and the smaller the value, the greater the difference of each spatial unit. If the value is 0, the situation is subject to random distribution.

### 2.3. Generalized LMDI Method

For further analysis of the SO_2_ emissions drivers, this paper adopts a GLMDI decomposition method based on an extended Kaya identity [32]. The SO_2_ emission ($SE^{t}$) in period *t* can be expressed as follows.

(3) $$SE^{t}=\underset{i}{\sum }SE_{i}^{t}=\underset{i}{\sum }\frac{GRP_{i}^{t}}{GDP^{t}}\times \frac{I_{i}^{t}}{GRP_{i}^{t}}\times \frac{E_{i}^{t}}{I_{i}^{t}}\times \frac{P_{i}^{t}}{E_{i}^{t}}\times \frac{C_{i}^{t}}{P_{i}^{t}}\times \frac{IN_{i}^{t}}{C_{i}^{t}}\times \frac{WG_{i}^{t}}{IN_{i}^{t}}\times \frac{SE_{i}^{t}}{WG_{i}^{t}}\times GDP^{t}\;=\underset{i}{\sum }Q_{i}^{t}\times SI_{i}^{t}\times EI_{i}^{t}\times EE_{i}^{t}\times UR_{i}^{t}\times FA_{i}^{t}\times WI_{i}^{t}\times SR_{i}^{t}\times GDP^{t}$$

where,$\;Q_{i}^{t}=\frac{GRP_{i}^{t}}{GDP^{t}}$, which is the proportion of the province *i*’s GRP ($GRP_{i}^{t}$) to the whole country ($GDP^{t}$), represents the economic growth level of province *i*. $SI_{i}^{t}=\frac{I_{i}^{t}}{GRP_{i}^{t}}$, which is the proportion of secondary sector of economy ($I_{i}^{t}$) to GRP ($GRP_{i}^{t}$), represents the industrial structure in province *i*. $UR_{i}^{t}=\frac{C_{i}^{t}}{P_{i}^{t}}$, which is the proportion of urban population ($C_{i}^{t}$) to total population ($P_{i}^{t}$), represents the urbanization rate of province *i.*$\;FA_{i}^{t}=\frac{IN_{i}^{t}}{C_{i}^{t}}$, which is the urban fixed assets investment ($IN_{i}^{t}$) per urban population ($C_{i}^{t}$), represents the intensity of urban fixed assets investment in province *i*.

$EI_{i}^{t}=\frac{E_{i}^{t}}{I_{i}^{t}}$, which is the ratio of energy consumption ($E_{i}^{t}$) to secondary industrial output ($I_{i}^{t}$) in province *i*, was used to denote the energy intensity. In China, energy consumption is mainly generated by the secondary industry. According to data from the National Bureau of Statistics (https://data.stats.gov.cn/easyquery.htm?cn=C01), in 2017, the energy consumption of the secondary industry in China was 294,488.04 × 10^4^ tons of coal equivalent (tce), while the consumption of the primary and thirdly industry were only 8931.23 × 10^4^ tce and 24,268.83 × 10^4^ tce, respectively. Therefore, to some extent, the more energy consumed per unit of the secondary industrial output, the greater the intensity of the province *i*’s energy consumption.

$EE_{i}^{t}=\frac{P_{i}^{t}}{E_{i}^{t}}$, is the ratio of population ($P_{i}^{t}$) to energy consumption ($E_{i}^{t}$) in province *i*. Commonly, industries sustain peoples’ lives by consuming energy to make products and generate income. At the same level of energy consumption, if industries in an area can sustain more people, that area is more energy efficient. The implication of this indicator is that the larger the population per unit of energy consumed, the more efficient the province *i*’s energy consumption.

$WI_{i}^{t}=\frac{WG_{i}^{t}}{IN_{i}^{t}}$, represents province *i*’s investment strength on waste gas treatment. $WG_{i}^{t}$ denotes the investment for industrial waste gas treatment projects. In China, this investment mainly concentrates on the old industrial pollution source treatment, such as desulfurization and denitrification. Old industrial pollution sources are mostly located in urban areas; the proportion of this investment to urban fixed assets investment ($IN_{i}^{t}$) was used to estimate the investment strength.

$SR_{i}^{t}=\frac{SE_{i}^{t}}{WG_{i}^{t}}$, is the ratio of SO_2_ emissions to investment on waste gas treatment projects in province *i*. The implication of this indicator is that the more the SO_2_ emissions per unit of investment, the higher the investment efficiency requirement in province *i*. Generally, the waste gas treatment investment varied from province to province, and the SO_2_ emissions of each province was also different. For provinces with high emissions but low investment, the pressure to reduce SO_2_ will be greater because of a lack of funds. For these provinces, the demand for investment efficiency will be higher. As a result, they are likely to increase the efficiency of their investment through various ways, such as technological innovation. Therefore, it is uncertain whether the higher requirement for investment efficiency will contribute to or inhibit SO_2_ emissions. To study this mechanism, the ratio of SO_2_ emissions to investment on waste gas treatment projects was adopted.

In order to assess the impact of capital and labor input on SO_2_ emissions, the C–D production function was introduced. The general formula of the C–D production function is as follows.

(4) $$GDP^{t}=A{\left(K^{t}\right)}^{\alpha }{\left(L^{t}\right)}^{\beta }$$

where, *A*, α, β are uncertain constant parameters, in general, *A*>0, 0<α<1, 0<β<1. *K* denotes capital input, measured by fixed asset investment. *L* denotes labor input, measured by the number of employed. *A* represents the level of technological progress to some extent but it was not considered in this study, see formula (18). Many methods have been used to calculate α and β of C–D production function, such as wage share in Gross Domestic Product (GDP), international experience reference, regression, etc. Among them, the regression method proved to be more scientific and accurate [33]. In the context of China’s rapid development, the parameter values over the years were likely to be different. In order to reflect this difference, the cross-sectional regression method was adopted in this paper to estimate the α and β of C–D function over the years, see Table 2, all the results were statistically significant.

The formula of GLMDI model is as follows:

(5) $$SE^{t}=\underset{i}{\sum }A\times Q_{i}^{t}\times SI_{i}^{t}\times EI_{i}^{t}\times EE_{i}^{t}\times UR_{i}^{t}\times FA_{i}^{t}\times WI_{i}^{t}\times SR_{i}^{t}\times {\left(K^{t}\right)}^{\alpha }\times {\left(L^{t}\right)}^{\beta }$$

The symbolism of each variable in GLMDI model is shown in Table 3.

Taking SO_2_ emissions in period 0 as the benchmark to investigate the change of SO_2_ emissions in period *t*, the summation decomposition of the GLMDI model is as follows.

(6) $$\begin{array}{ll} \Delta SE_{tot} & =SE^{t}-SE^{0}\\ & =\Delta SE_{K}^{t}+\Delta SE_{L}^{t}+\Delta SE_{Q}^{t}+\Delta SE_{SI}^{t}+\Delta SE_{EI}^{t}+\Delta SE_{EE}^{t}+\Delta SE_{UR}^{t}+\Delta SE_{FA}^{t}+\Delta SE_{WI}^{t}+\Delta SE_{SR}^{t} \end{array}$$

The calculation method of decomposition factors in formula (6) are as follows.

(7) ΔSEKt={0,SE0×SEt=0;∑iwiln((Kt)α(K0)α), SE0×SEt≠0; 

(8) ΔSELt={0,SE0×SEt=0;∑iwiln((Lt)β(L0)β), SE0×SEt≠0; 

(9) ΔSEQt={0,SE0×SEt=0;∑iwiln(QitQi0), SE0×SEt≠0; 

(10) ΔSESIt={0,SE0×SEt=0;∑iwiln(SIitSIi0), SE0×SEt≠0; 

(11) ΔSEEIt={0,SE0×SEt=0;∑iwiln(EIitEIi0), SE0×SEt≠0; 

(12) ΔSEEEt={0,SE0×SEt=0;∑iwiln(EEitEEi0), SE0×SEt≠0; . 

(13) ΔSEURt={0,SE0×SEt=0;∑iwiln(URitURi0), SE0×SEt≠0; 

(14) ΔSEFAt={0,SE0×SEt=0;∑iwiln(FAitFAi0), SE0×SEt≠0; 

(15) ΔSEWIt={0,SE0×SEt=0;∑iwiln(WIitWIi0), SE0×SEt≠0; 

(16) ΔSESRt={0,SE0×SEt=0;∑iwiln(SRitSRi0), SE0×SEt≠0; 

where,

(17) $$w_{𝒾}=\frac{SE_{𝒾}^{t}-SE_{𝒾}^{0}}{\ln SE_{𝒾}^{t}-\ln SE_{𝒾}^{0}}$$

For A is a constant,

(18) $$\ln\left(A^{t}/A^{0}\right)=0$$

This study gave no consideration on it. National data, such as GDP, fixed asset investments, employed numbers, and provincial data, such as GRP, industrial output, fixed asset investments, total population, urban population (2005–2017), and investment in industrial waste gas treatment projects (2004–2017) were all derived from the National Bureau of Statistics of China. The urban population from 2000 to 2004 were acquired from the China Compendium of Statistics 1949–2008. Investment in industrial waste gas treatment projects from 2000 to 2003 were obtained from the China Statistical Yearbook (2001–2004). Provincial number of employed in urban areas were from the Economy Prediction System (EPS) database. Data of total energy consumption were from the China Energy Statistical Yearbook (2001–2018). Interpolation had been used to fill a small amount of missing data. The data of GDP and industrial output value in this paper had been converted to comparable prices based on the year 2000.

## 3. Results and Discussion

### 3.1. Spatial-Temporal Analysis of (De)Coupling Conditions

According to the results of decoupling indexes assessment, as shown in Table 1, there were five decoupling states within the scope of this study, namely, strong decoupling (SD), weak decoupling (WD), expansive negative decoupling (END), expansive coupling (EC), and recessive coupling (RC), sorting from most to least. Based on the decoupling state classification, see Table 1, SD is the most desirable condition where SO_2_ emissions decline along with economic development. Therefore, the frequent emergence of SD indicated that the overall decoupling status of provinces in China was favorable.

**Figure 1:**
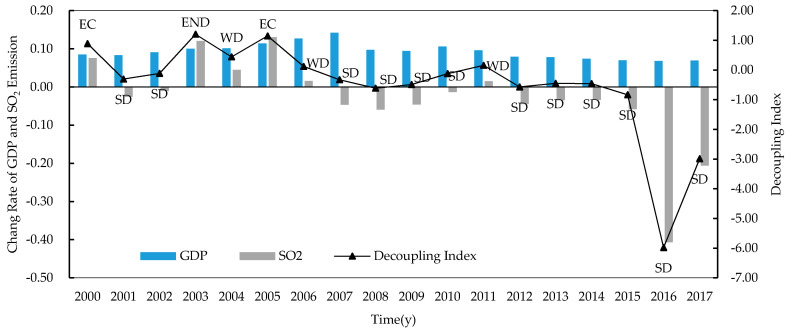
Decoupling conditions in China 2000–2017.

From the perspective of temporal contrast, as shown in Figure 1, the overall decoupling condition in China was improving. At the beginning of the 21st country, the GDP and SO_2_ emission went up together, showing the characteristics of EC. Then, the decoupling situation was unstable from 2001 to 2011 for the fluctuation of SO_2_ emission change rates. With economic growth consistently positive, the change rate of SO_2_ emissions had been negative since 2012, so that the decoupling condition had remained to SD. It is worth noting that significant decline of SO_2_ emissions occurred in 2016 and 2017, which showed the performance of China’s vigorous efforts to combat air pollution in recent years; those efforts consolidated the achievement of reaching SD.

However, the situation varies from province to province; see Table 4. From 2001 to 2014, there were many changes and fluctuations of the decoupling state. After 2015, 28 provinces reached the SD state, accounting for 93.3% of the provinces studied. Among all the administrative units, Beijing was in the best state, which had almost achieved SD in all periods within this study, except 2004. This situation shows that Beijing’s decoupling condition had been relatively stable.

On the contrary, some provinces should be focused on. As shown in Table 4, Guizhou was in WD scenario in 2017, where the SO_2_ emissions grows more slowly than the economy, which was not the best, but an acceptable condition. The condition in Liaoning became RD in 2016, which means the economy was in recession while the SO_2_ emissions decreased even more significantly. Although this condition was good from an environmental point of view, emission reduction at the cost of economic recession was not acceptable. To achieve the goal of sustainable development, the situation in Liaoning province needed to be carefully considered. Moreover, Hainan, Qinghai, and Xinjiang had the fewest SD states among the provinces studied, only six times from 2001 to 2017, which means their situations were precarious and complicated in former years. However, these three provinces reached SD state after 2015, which suggested that their situations were expected to gradually stabilize. For these provinces, recent experience could be referenced and efforts should be made to consolidate their SD situations.

**Figure 2:**
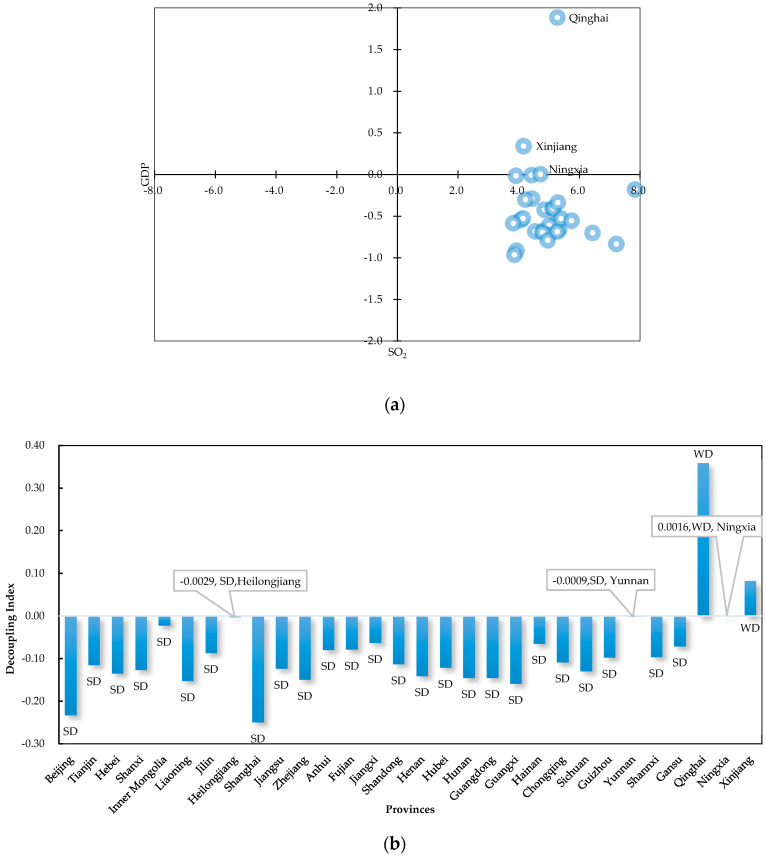
Overall decoupling conditions of 30 provinces in China. (**a**) The change rate of Gross Regional Product (GRP) and SO_2_ emissions. (**b**) The overall decoupling indexes.

Figure 2a—from the perspective of spatial analysis. In the context of positive economic growth in all provinces, SO_2_ emissions increased in only three provinces (Qinghai, Xinjiang, and Ningxia) and fell in all others, so that Qinghai, Xinjiang, and Ningxia failed to achieve SD condition; see Figure 2b.

**Figure 3:**
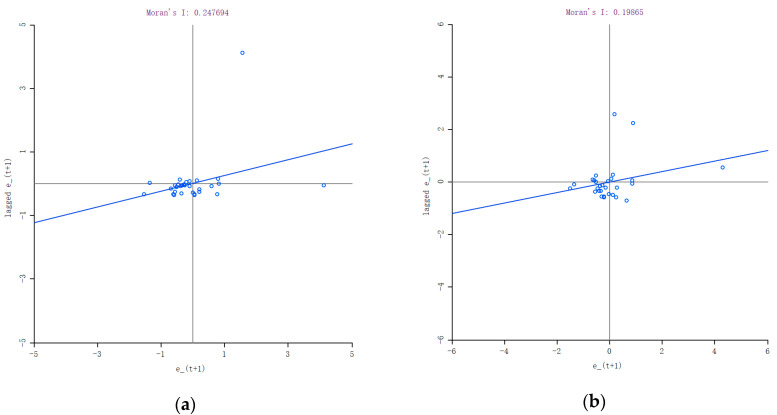
Moran’s Index scatter plot of decoupling index and its significance test. (**a**) Moran’s I scatter plot of 30 provinces. (**b**) Moran’s I scatter plot for robust check.

The Moran Index scatter plot was used to further assess the spatial relationship among provinces, as shown in Figure 3a. The *x*-axis represents the normalized provincial decoupling index ($e_{t+1}$) and the *y*-axis represents the lagged value, that is, the normalized $e_{t+1}$ of adjacent units of each province. The diagonal in the graph can be regarded as the linear fitting of the scatters. The Moran’s I is the slope of the diagonal, which is greater than 0, representing positive spatial autocorrelation. This result was permutated 999 times to test its significance and the *p* value was 0.001, indicating at least 99% certainty that the results were significant. Moreover, it is worth noting that there is an upper-right outlier in Figure 3a. To exclude its influence on the result, Moran’s I was recalculated after removing the outlier for robust check, as shown in Figure 3b. The recalculated Moran’s I was still greater than 0, and its *p* value after 999 times permutating was 0.028, which was lower than 0.05, indicating that the results were significant at least 95% certainty. That is, generally, the outlier did not have a decisive influence on the results; the decoupling conditions of neighboring provinces would affect each other.

To give a more comprehensive analysis of spatial relationships between provinces, Figure 3a takes all provinces into account. Specific to each province’s situation, the four quadrants identify four kinds of spatial relationships and they can be further categorized into two groups: positive and negative spatial autocorrelation [34]. Provinces in the first and third quadrants, respectively, exhibited high–high (H–H) and low–low (L–L) aggregation, indicating that these provinces tended to be adjacent to provinces with similar decoupling indexes, that is, positively autocorrelated. Provinces in the second and fourth quadrants exhibited low–high (L–H) and high–low (H–L) aggregation separately, indicating that these provinces tended to be adjacent to provinces with opposite decoupling indexes, which is called negative autocorrelation. The map of Local Indications of Spatial Association (LISA) aggregation was applied to analyze the spatial correlation and the significance of each province. The LISA is a space-based statistical technique; it gives an indication of the extent to which a significant spatial clustering of homogeneous values existing around a particular observation [35,36]. The results of LISA, which indicate spatial correlation of decoupling index in 30 provinces, can be calculated through software GeoDa, as shown in Table 5.

Four provinces, Xinjiang, Jiangxi, Anhui, and Fujian showed significantly positive autocorrelation. Cross-regional coordination could be considered in these regions. Among these provinces, Xinjiang did not, overall, reach SD state; see Figure 2. Its conditions could have a bad effect on neighboring provinces. Therefore, in order to maintain favorable decoupling status, the provinces adjacent to Xinjiang should give Xinjiang the necessary assistance to make it achieve SD faster. Moreover, there were four provinces in states of significantly negative autocorrelation, Shanghai, Jiangsu, Zhejiang, and Shaanxi; they were all in H–L condition. All of these provinces had, overall, reached SD state; see Figure 2. However, their developments might have dampening effects on the surrounding areas. For these provinces, there might be trade-offs with their neighbors. Therefore, these provinces should learn the decoupling trends of neighbors and complement each other when seeking self-development.

### 3.2. Driving Factors Decomposition of SO_2_ emissions

Nevertheless, the understanding of decoupling conditions alone cannot maximize the efficiency of SO_2_ reduction. Therefore, panel data of 30 provinces from 2000 to 2017 were used to identify the effect of each driving factor on SO_2_ emissions. Decomposition and analysis were carried out according to Equations (7)–(17). For the whole country, decomposition factors were calculated, taking the year 2000 as the benchmark; the results are shown in Table 6.

In general, the impact of elements on SO_2_ emissions was negative within this study; see row $\;\Delta SE_{tot}$, indicating that SO_2_ emissions had been overall suppressed in China from 2000 to 2017. The driving factors can be categorized into two groups: the positive factors, which would cause emissions increase, and the negative factors, which could facilitate emissions reduction. As shown in Table 6, the capital ($\Delta SE_{K}^{t}$), the labor ($\Delta SE_{L}^{t}$), the economic growth level ($\Delta SE_{Q}^{t}$), the proportion of secondary sector of economy to GRP ($\Delta SE_{SI}^{t}$), the urbanization rate ($\Delta SE_{UR}^{t}$) and the fixed assets investment ($\Delta SE_{FA}^{t}$) were positive drivers. Obviously, all of these positive drivers are important factors to promote social and economic development, which means that China’s socio-economic development, indeed, led to an increase in SO_2_ emissions.

On the contrary, the energy consumption intensity ($\Delta SE_{EI}^{t}$), the energy efficient ($\Delta SE_{EE}^{t}$), the waste gas treatment investment ($\Delta SE_{WI}^{t}$), and the investment efficiency requirement ($\Delta SE_{SR}^{t}$) were negative drivers. The first two negative factors are related to energy consumption, which shows that China’s energy management has played a beneficial role in reducing SO_2_ emissions. The negative impact of these energy factors is favorable for China, because China remained the world’s largest energy consumer in 2017, accounting for 23.2% of global energy consumption and 33.6% of global energy consumption growth, according to the 2018 British Petroleum (BP) World Energy Statistical Yearbook (http://www.199it.com/archives/767423.html). As China develops further, it will be difficult for its energy consumption to decline in short-term. Therefore, energy management will continue to be important to SO_2_ emissions reduction; the inhibitory effect of energy factors can be good to China’s atmospheric environment protection in the long run. The latter two negative factors are related to investment in waste gas treatment projects, indicating that China’s investment in waste gas treatment has made achievement in reducing SO_2_ emissions. The most significant negative driver is $\Delta SE_{SR}^{t}$, which denotes the requirement of waste gas reduction investment efficiency. In order to meet the investment efficiency requirement, the Chinese government and enterprises accelerated technological innovation of SO_2_ emission reduction and adopted a series of mandatory emission reduction policies. At the second National Conference on Environmental Science and Technology of China, the minister of Ministry of Environmental Protection (now the Ministry of Ecology and Environment) said that technological progress accounted for 66% of sulfur dioxide emissions reduction (http://www.cinic.org.cn/zgzz/cx/136582.html). Moreover, China issued at least 237 air pollution control regulations at the national level from 2000 to 2017 (data were obtained from the websites of the Ministry of Ecology and Environment (http://www.mee.gov.cn/), Ministry of Finance (http://www.mof.gov.cn/index.htm), Resource Conservation and Environmental Protection division of National Development and Reform Commission (https://www.ndrc.gov.cn/fzggw/jgsj/hzs/), and the Laws and Regulations Database of Peking University), which urged and guided the emission reduction of SO_2_ and other air pollutants. All of these reasons made $\Delta SE_{SR}^{t}$ become the most important emission reduction driver.

However, only a macro analysis of the overall situation of China cannot reveal the changes of each factor over the years. For a rapidly developing country, the effects of each factor are likely to be different at different development stages. Therefore, the analysis of the changes in the impact of various factors in different years will provide a more specific reference for China’s SO_2_ emission reduction. To compare the changes of decomposition factors over the years, this study calculated each decomposition factor taking the previous year as the benchmark, the results are shown in Table 7. 

From the perspective of total effect, as shown in column $\Delta SE_{tot}$, the impact of elements on SO_2_ emissions was negative only expect that from year 2002 to 2003 and from year 2004 to 2005, indicating that SO_2_ emissions suppression in China was relatively stable. The overall positive effect of elements in 2002–2003 and 2004–2005 had leaded to significant increase of SO_2_ emissions in China; see Figure 1, which was the result of multifactorial interaction. To find out the key factors, as shown in row 2002–2003, the positive effect of the fixed assets investment per person ($\Delta SE_{FA}^{t}$) was relatively high, while the negative effect of the energy intensity ($\Delta SE_{EI}^{t}$) was the lowest compared to other periods. In other words, the fixed assets investment per person significantly contributed to SO_2_ emissions increase, while the industrial energy intensity control did not have adequate restraining effect on SO_2_ emissions from 2002 to 2003. From 2004 to 2005, the urbanization rate ($\Delta SE_{UR}^{t}$) was much greater than that in other periods, while the contribution of $\Delta SE_{EI}^{t}$ was still relatively low, which means that rapid urbanization contributed significantly to SO_2_ emissions while the inhibition effect of industrial energy intensity control on SO_2_ was relatively weak compared with other stages.

From the perspective of positive factorization, there were two factors that always behaved as positive drivers, the labor ($\Delta SE_{L}^{t}$) and the economic growth level ($\Delta SE_{Q}^{t}$), indicating that labor and economic development always contributed to higher SO_2_ emissions in China. Besides, the capital ($\Delta SE_{K}^{t}$), the proportion of secondary sector of economy to GRP ($\Delta SE_{SI}^{t}$), the urbanization rate ($\Delta SE_{UR}^{t}$), and the fixed assets investment per person ($\Delta SE_{FA}^{t}$) exhibited acceleration impact on SO_2_ emissions in no less than 14 years. Among all the positive drivers, the labor ($\Delta SE_{L}^{t}$), the economic growth level ($\Delta SE_{Q}^{t}$), the capital ($\Delta SE_{K}^{t}$), the urbanization rate ($\Delta SE_{UR}^{t}$), and the fixed assets investment per person ($\Delta SE_{FA}^{t}$) are key indicators of national progress, and cannot be suppressed just for the sake of SO_2_ emission reduction. While, the proportion of secondary sector of economy to GRP can be weighed in the future. In fact, the adjustment of economic structure has been attached great importance by the Chinese government, and great breakthroughs have been made in the past period of time. However, according to data from the World Bank World Development Indicator (WDI) database, in 2017, the proportion of secondary industry was 40.5% in China, while this proportion was less than 30% in developed countries, such as the United States, Japan, and Canada, which means that China’s economic structure still has potential for optimization. To reduce the proportion of secondary industry and, thus, its impact on SO_2_ emissions, the Chinese government should continue to encourage non-industrial enterprises to drive the economy. For example, the government could sequentially improve the proportion of primary and tertiary industry in GRP to optimize the structure of enterprises in China.

From the perspective of negative factorization, the intensity ($\Delta SE_{EI}^{t}$) and the efficiency ($\Delta SE_{EE}^{t}$) of energy consumption had suppression impact on SO_2_ emissions in 16 years, and they all remained negative after year 2013, indicating that the inhibition of SO_2_ emissions by energy management was stable over years, which is beneficial for China, referring to the macro analysis of the whole country above. For the waste gas treatment investment ($\Delta SE_{WI}^{t}$), and the investment efficiency requirement ($\Delta SE_{SR}^{t}$), the conditions before 2015 were unstable, while these two factors remained negative from 2015 to 2017. Whether they will change next is still uncertain; thus, the government and enterprises need to take necessary measures to stabilize their negative influence.

### 3.3. Limitations

Due to the lack of public data, some factors affecting sulfur dioxide emissions, such as indicators measuring the development of SO_2_ filtration technology, were not taken into account in the model. This issue also made some indicators be not straightforward. As there is no available public data on investments specifically for SO_2_ treatment, the investment in industrial waste gas treatment projects was used to estimate the investment strength of waste gas treatment and the investment efficiency requirement. However, the waste gas projects mainly include desulfurization and denitrification, they do not solely consider SO_2_. In addition, at the provincial level, comprehensive energy consumption data for the secondary industry are not directly disclosed in China. For China’s energy consumption is mainly generated by the secondary industry, the total energy consumption, instead of the energy consumption for the secondary industry, was used to measure the energy intensity and efficiency.

Moreover, this paper used the data of each province in China to study the factorization of the whole country, and the analysis was relatively macro, while the situation of each province was different, so the factorization of each province would be more targeted. However, according to the principle of the GLMDI model, more microscopic data are needed to realize the factor decomposition at the provincial level, such as the data of cities within the jurisdiction of each province. At present, some data of key driving factors, such as the investment data of waste gas treatment, are not publicly available at the city level, which limits further refinement of the study. In future research, further optimization of index selection can be considered, and different theoretical models can be tried to achieve the impact factor decomposition at the provincial level.

## 4. Conclusions

According to the spatial-temporal analysis of (de)coupling condition, China’s decoupling scenario had become better from 2000 to 2017. Over the entire time period, Qinghai, Xinjiang, and Ningxia failed to achieve SD condition. The overall relationship between SO_2_ emission and economic growth had achieved the SD stage since 2012. Provinces, except Liaoning and Guizhou, had all reached SD state since 2015. Among the provinces, Beijing has the most stable decoupling condition, which can provide reference for other provincial administrative units. While, Liaoning and Guizhou need to be paid more attention to by the Chinese government in the future, as their decoupling situations were still unstable in recent years. The decoupling indexes of neighboring provinces indicated significant spatial dependence at more than 95% certainty. Coordinated development across provinces could be taken into account in Xinjiang, Jiangxi, Anhui, and Fujian. In particular, the provinces adjacent to Xinjiang should give Xinjiang the necessary assistance for maintaining their own SD state. Complementary development with neighboring provinces should be considered in Shanghai, Jiangsu, Zhejiang, and Shaanxi.

According to the driving factors decomposition, as a whole, SO_2_ emissions suppression in China was relatively stable, while the driving factors demonstrated different impacts on SO_2_ emissions over the years. Thus, China should examine the influence of various factors from a dynamic perspective to make correct decisions in SO_2_ emission reduction. The positive driver that should be paid most attention to is the proportion of the secondary sector of the economy to GRP. This is not only because of the adjustment potential of this driver, but also because optimizing this factor to reduce SO_2_ emissions will not be at the expense of the country’s socio-economic development. The main negative drivers were related to energy consumption and investment in waste gas treatment. The inhibitory effect of energy factors can be beneficial to China’s SO_2_ emissions reduction in the long run. Moreover, the negative drivers that should be focused on are waste gas treatment investment and investment efficiency requirement, because their effects are still uncertain. To stabilize the negative effect of these factors, the direction of investment in waste gas treatment should be optimized. The government should focus on the support of low-cost and high-efficiency technologies for SO_2_ reduction. The enterprises should speed up the elimination of backward production and invest more in environmentally friendly production.

## Figures and Tables

**Table 1:** Decoupling State Classification.

Conditions		$\Delta G_{t+1}$	$\Delta S_{t+1}$	$e_{t+1}$	Characterization
Negative decoupling	Strong negative decoupling (SND)	<0	>0	$e_{t+1}$ < 0	Economic recession along with intensified pollution
	Weak negative decoupling (WND)	<0	<0	0 $\leq \;e_{t+1}$ < 0.8	Fast economic recession along with slow pollution decline
	Expansive negative decoupling (END)	>0	>0	$e_{t+1}$ > 1.2	Slow economy growth with fast intensified pollution
Coupling	Recessive coupling (RC)	<0	<0	0.8 $\leq e_{t+1}\leq$ 1.2	The economy and pollution go down together
	Expansive coupling (EC)	>0	>0	0.8 $\leq e_{t+1}\leq$ 1.2	The economy and pollution go up together
Decoupling	Recessive decoupling (RD)	<0	<0	$e_{t+1}$ > 1.2	Slow economic recession with significantly pollution reduction
	Weak decoupling (WD)	>0	>0	0 $\leq e_{t+1}$ < 0.8	Fast economic growth with slow pollution increase
	Strong decoupling (SD)	>0	<0	$e_{t+1}$ < 0	Economic growth along with pollution reduction

**Table 2:** Parameters of the Cobb–Douglas (C–D) function from 2000 to 2017.

Years	α	β
2000	0.745 **	0.459 **
2001	0.728 **	0.482 **
2002	0.728 **	0.474 **
2003	0.694 **	0.505 **
2004	0.651 **	0.555 **
2005	0.583 **	0.618 **
2006	0.575 **	0.618 **
2007	0.578 **	0.610 **
2008	0.450 **	0.721 **
2009	0.378 *	0.802 **
2010	0.461 **	0.708 **
2011	0.407 *	0.760 **
2012	0.411 *	0.751 **
2013	0.424 **	0.698 **
2014	0.515 **	0.599 **
2015	0.604 **	0.501 **
2016	0.622 **	0.481 **
2017	0.699 **	0.411 **

* Indicates *p* value ≤ 0.05; ** indicates *p* value ≤ 0.01.

**Table 3:** Meaning of variables in the generalized logarithmic mean Divisia index (GLMDI) model.

Variable	Meaning	Unit
$Q_{i}^{t}$	The economic growth level of province *i*.	%
$SI_{i}^{t}$	The industrial structure in province *i*.	%
$EI_{i}^{t}$	The energy consumption intensity in province *i*.	tce/10^4^ RMB
$EE_{i}^{t}$	The energy consumption efficiency in province *i.*	Person/tce
$UR_{i}^{t}$	The urbanization rate in province *i.*	%
$FA_{i}^{t}$	The intensity of urban fixed assets investment in province *i.*	10^4^ RMB/Person
$WI_{i}^{t}$	The investment strength of waste gas treatment in province *i*.	%
$SR_{i}^{t}$	The investment efficiency requirement of waste gas treatment in province *i*.	t/10^4^ RMB
$GDP^{t}$	Gross Domestic Product in year *t*	10^8^ RMB
$GRP_{i}^{t}$	Gross Regional Product of province *i* in year *t*	10^8^ RMB
$I_{i}^{t}$	Secondary industrial output value of province *i* in year *t*	10^8^ RMB
$E_{i}^{t}$	Total energy consumption of province *i* in year *t*	10^4^ tce
$P_{i}^{t}$	Total population of province *i* in year *t*	10^4^ people
$C_{i}^{t}$	Urban population of province *i* in year *t*	10^4^ people
$IN_{i}^{t}$	Urban fixed assets investment of province *i* in year *t*	10^8^ RMB
$WG_{i}^{t}$	Investment in industrial waste gas treatment projects of province *i* in year *t*	10^8^ RMB
$SE_{i}^{t}$	SO_2_ emissions of province *i* in year *t*	10^4^ t

tce: tons of coal equivalent.

**Table 4:** Annual decoupling condition of each province.

Category	Province	2001	2002	2003	2004	2005	2006	2007	2008	2009	2010	2011	2012	2013	2014	2015	2016	2017
**Provinces in favorable stages**	Beijing	SD	SD	SD	WD	SD	SD	SD	SD	SD	SD	SD	SD	SD	SD	SD	SD	SD
	Tianjin	SD	SD	WD	SD	EC	SD	SD	SD	SD	SD	SD	SD	SD	SD	SD	SD	SD
	Hebei	SD	SD	EC	WD	WD	WD	SD	SD	SD	SD	END	SD	SD	SD	SD	SD	SD
	Shanxi	SD	WD	EC	WD	WD	SD	SD	SD	SD	SD	EC	SD	SD	SD	SD	SD	SD
	Inner Mongolia	SD	EC	END	SD	EC	WD	SD	SD	SD	SD	WD	SD	SD	SD	SD	SD	SD
	Jilin	SD	WD	WD	WD	END	WD	SD	SD	SD	SD	EC	SD	SD	SD	SD	SD	SD
	Heilongjiang	SD	SD	END	WD	END	WD	SD	SD	SD	SD	WD	SD	SD	SD	SD	SD	SD
	Shanghai	WD	SD	WD	WD	WD	SD	SD	SD	SD	SD	SD	SD	SD	SD	SD	SD	SD
	Jiangsu	SD	SD	WD	SD	WD	SD	SD	SD	SD	SD	WD	SD	SD	SD	SD	SD	SD
	Zhejiang	SD	WD	EC	WD	WD	SD	SD	SD	SD	SD	SD	SD	SD	SD	SD	SD	SD
	Anhui	WD	WD	END	WD	END	WD	SD	SD	SD	SD	SD	SD	SD	SD	SD	SD	SD
	Fujian	SD	SD	END	WD	END	WD	SD	SD	SD	SD	SD	SD	SD	SD	SD	SD	SD
	Jiangxi	SD	SD	END	END	END	WD	SD	SD	SD	SD	WD	SD	SD	SD	SD	SD	SD
	Shandong	SD	SD	WD	SD	WD	SD	SD	SD	SD	SD	END	SD	SD	SD	SD	SD	SD
	Henan	WD	WD	EC	END	END	WD	SD	SD	SD	SD	WD	SD	SD	SD	SD	SD	SD
	Hubei	SD	SD	END	END	WD	WD	SD	SD	SD	SD	WD	SD	SD	SD	SD	SD	SD
	Hunan	SD	SD	END	WD	WD	WD	SD	SD	SD	SD	SD	SD	SD	SD	SD	SD	SD
	Guangdong	WD	WD	WD	WD	EC	SD	SD	SD	SD	SD	SD	SD	SD	SD	SD	SD	SD
	Guangxi	SD	SD	END	WD	WD	SD	SD	SD	SD	SD	SD	SD	SD	SD	SD	SD	SD
	Chongqing	SD	SD	EC	WD	WD	WD	SD	SD	SD	SD	SD	SD	SD	SD	SD	SD	SD
	Sichuan	SD	SD	WD	WD	WD	SD	SD	SD	SD	SD	SD	SD	SD	SD	SD	SD	SD
	Yunnan	SD	WD	END	WD	EC	WD	SD	SD	SD	WD	END	SD	SD	SD	SD	SD	SD
	Shaanxi	SD	WD	END	WD	EC	WD	SD	SD	SD	SD	END	SD	SD	SD	SD	SD	SD
	Gansu	WD	END	END	SD	END	SD	SD	SD	SD	EC	EC	SD	SD	WD	SD	SD	SD
	Ningxia	SD	EC	END	WD	END	EC	SD	SD	SD	SD	END	SD	SD	SD	SD	SD	SD
**Provinces in need of attention**	Liaoning	SD	SD	WD	WD	END	WD	SD	SD	SD	SD	EC	SD	SD	SD	SD	RD	SD
	Guizhou	SD	SD	SD	SD	WD	WD	SD	SD	SD	SD	SD	SD	SD	SD	SD	SD	WD
	Hainan	WD	EC	WD	WD	SD	WD	WD	SD	WD	END	EC	WD	SD	WD	SD	SD	SD
	Qinghai	EC	SD	END	END	END	WD	WD	WD	WD	WD	WD	SD	WD	SD	SD	SD	SD
	Xinjiang	SD	SD	EC	END	WD	WD	WD	WD	WD	SD	END	WD	WD	WD	SD	SD	SD

**Table 5:** Spatial correlation of decoupling index in 30 provinces.

Quadrant	Spatial Correlation	Provinces
I	H–H	Xinjiang *, Gansu, Yunnan
II	L–H	Beijing, Sichuan, Guizhou, Tianjin
III	L–L	Inner Mongolia, Heilongjiang, Jiangxi *, Hainan, Anhui **, Qinghai, Fujian *, Ningxia
IV	H–L	Hebei, Shanxi, Liaoning, Jilin, Shanghai **, Jiangsu *, Zhejiang **, Shandong, Henan *, Hubei, Hunan, Guangdong, Guangxi, Chongqing, Shannxi

* indicates *p* value ≤0.05; ** indicates *p* value ≤0.01.

**Table 6:** The overall influencing factors decomposition in China.

Indicators	2000–2017
The capital input ($\Delta SE_{K}^{t}$)	334.4
The labor input ($\Delta SE_{L}^{t}$)	40.3
The economic growth level ($\Delta SE_{Q}^{t}$)	350.2
The industrial structure ($\Delta SE_{SI}^{t}$)	279.8
The energy intensity ($\Delta SE_{EI}^{t}$)	−1052.9
The energy efficiency ($\Delta SE_{EE}^{t}$)	−1460.1
The urbanization rate ($\Delta SE_{UR}^{t}$)	638.6
The fixed assets investment ($\Delta SE_{FA}^{t}$)	3264.4
The waste gas treatment investment ($\Delta SE_{WI}^{t}$)	−2050.8
The investment efficiency requirement ($\Delta SE_{SR}^{t}$)	−3061.4
The total effect ($\Delta SE_{tot}$)	−2717.5

**Table 7:** The annual contribution of driving factors to SO_2_ emissions.

Periods	$\mathrm{The}\;\mathrm{Capital}\;\mathrm{Input}\;(\Delta SE_{K}^{t})$	$\mathrm{The}\;\mathrm{Labor}\;\mathrm{Input}\;\left(\Delta SE_{L}^{t}\right)$	$\mathrm{The}\;\mathrm{Economic}\;\mathrm{Growth}\;\mathrm{Level}\;\left(\Delta SE_{Q}^{t}\right)$	$\mathrm{The}\;\mathrm{Industrial}\;\mathrm{Structure}\;(\Delta SE_{SI}^{t})$	$\mathrm{The}\;\mathrm{Energy}\;\mathrm{Intensity}\;\left(\Delta SE_{EI}^{t}\right)$	$\mathrm{The}\;\mathrm{Energy}\;\mathrm{Efficiency}\;(\Delta SE_{EE}^{t})$	$\mathrm{The}\;\mathrm{Urbanization}\;\mathrm{Rate}\;(\Delta SE_{UR}^{t})$	$\mathrm{The}\;\mathrm{Fixed}\;\mathrm{Assets}\;\mathrm{Investment}\;\left(\Delta SE_{FA}^{t}\right)$	$\mathrm{The}\;\mathrm{Waste}\;\mathrm{Gas}\;\mathrm{Investment}\;\mathrm{Treatment}\;\left(\Delta SE_{WI}^{t}\right)$	$\mathrm{The}\;\mathrm{Investment}\;\mathrm{Efficiency}\;\mathrm{Requirement}\;\left(\Delta SE_{SR}^{t}\right)$	$\mathrm{The}\;\mathrm{Total}\;\mathrm{Effect}\;\left(\Delta SE_{tot}\right)$
2000–2001	5.6	9.1	20.6	7.6	−72.5	−99.6	47.8	211.6	−920.7	570.5	−220.1
2001–2002	2.5	5.3	23.1	29.0	−65.7	−123.9	59.9	198.9	−449.4	172.4	−147.9
2002–2003	30.4	6.3	40.0	65.2	−23.0	−260.4	−23.9	545.3	−84.8	−167.9	127.1
2003–2004	77.1	8.6	69.1	58.0	122.4	−445.1	71.9	460.2	754.1	−1203.2	−26.7
2004–2005	22.2	7.6	41.4	58.1	−35.8	−329.4	153.2	433.1	44.9	−330.3	65.0
2005–2006	20.6	6.6	13.2	68.5	−132.1	−219.6	62.8	442.6	−10.5	−474.9	−222.9
2006–2007	55.9	7.0	12.2	55.3	−162.8	−224.7	67.3	505.2	−92.0	−614.6	−391.3
2007–2008	91.9	5.6	56.6	25.7	−162.2	−124.6	68.4	481.7	−615.9	−98.2	−271.0
2008–2009	−20.8	6.3	51.5	29.3	−152.2	−117.5	54.5	548.7	−906.2	181.2	−325.0
2009–2010	35.8	5.7	56.3	63.4	−148.3	−177.3	86.9	380.9	−951.2	438.4	−209.5
2010–2011	57.2	6.9	58.7	47.5	−116.3	−180.9	64.4	224.4	77.2	−343.5	−104.5
2011–2012	9.7	6.0	60.2	27.7	−135.3	−105.9	64.3	353.4	−129.1	−400.4	−249.3
2012–2013	2.6	5.2	37.6	15.7	−282.6	84.0	46.7	332.4	1739.8	−2203.4	−222.1
2013–2014	5.2	4.3	16.2	4.4	−100.7	−52.9	47.3	229.3	−12.6	−343.8	−203.4
2014–2015	−21.0	2.5	11.7	−20.8	−87.1	−21.9	47.6	90.3	−1065.8	801.0	−263.6
2015–2016	−5.4	1.4	5.1	−16.6	−57.5	−16.8	36.3	13.2	−81.4	−733.4	−855.2
2016–2017	38.7	0.2	3.5	−11.1	−31.8	−20.2	23.2	−30.5	−190.1	−35.8	−253.9
